# SACFIR: SDN-Based Application-Aware Centralized Adaptive Flow Iterative Reconfiguring Routing Protocol for WSNs

**DOI:** 10.3390/s17122893

**Published:** 2017-12-13

**Authors:** Muhammad Aslam, Xiaopeng Hu, Fan Wang

**Affiliations:** School of Computer Science and Technology, Dalian University of Technology, Dalian 116000, China; aslamhayat@mail.dlut.edu.cn (M.A.); wangfan@dlut.edu.cn (F.W.)

**Keywords:** software-defined networking, wireless sensor networks, flow reconfiguring, routing, application-specific, heterogeneity awareness

## Abstract

Smart reconfiguration of a dynamic networking environment is offered by the central control of Software-Defined Networking (SDN). Centralized SDN-based management architectures are capable of retrieving global topology intelligence and decoupling the forwarding plane from the control plane. Routing protocols developed for conventional Wireless Sensor Networks (WSNs) utilize limited iterative reconfiguration methods to optimize environmental reporting. However, the challenging networking scenarios of WSNs involve a performance overhead due to constant periodic iterative reconfigurations. In this paper, we propose the SDN-based Application-aware Centralized adaptive Flow Iterative Reconfiguring (SACFIR) routing protocol with the centralized SDN iterative solver controller to maintain the load-balancing between flow reconfigurations and flow allocation cost. The proposed SACFIR’s routing protocol offers a unique iterative path-selection algorithm, which initially computes suitable clustering based on residual resources at the control layer and then implements application-aware threshold-based multi-hop report transmissions on the forwarding plane. The operation of the SACFIR algorithm is centrally supervised by the SDN controller residing at the Base Station (BS). This paper extends SACFIR to SDN-based Application-aware Main-value Centralized adaptive Flow Iterative Reconfiguring (SAMCFIR) to establish both proactive and reactive reporting. The SAMCFIR transmission phase enables sensor nodes to trigger direct transmissions for main-value reports, while in the case of SACFIR, all reports follow computed routes. Our SDN-enabled proposed models adjust the reconfiguration period according to the traffic burden on sensor nodes, which results in heterogeneity awareness, load-balancing and application-specific reconfigurations of WSNs. Extensive experimental simulation-based results show that SACFIR and SAMCFIR yield the maximum scalability, network lifetime and stability period when compared to existing routing protocols.

## 1. Introduction

Modern dramatic progression in Micro Electro-Mechanical Systems (MEMS) has enabled Wireless Sensor Networks (WSNs) to support a wide range of military and civil applications that demand real-time environmental monitoring, infrastructure protection and smart network performance analysis. Successful online route-establishing traffic engineering in WSNs guarantees reliable network performance, but it also faces many challenges due to the dynamic nature of the network topology, limited battery life, lack of a conventional addressing scheme and higher distributed self-organization of sensor nodes [1,2,3,4]. Conventional cluster-based organizations of WSNs formulate iterative traffic engineering to compute online network reconfigurations to establish stable routes. Frequent network reconfigurations offer theoretically better Quality of Service (QoS), but they also demand global knowledge of the network topology and other available resources [3,5,6,7].

The existing challenging task of ensuring the commercial success of WSNs needs attention from researchers to provide real-time installation and establish adaptive reconfigurable management. The proposed application-specific requirements of network operators demand flexible networking scenarios and the ability to recycle the limited network resources. Recent research on WSNs highlights some solutions to deal with the challenges encountered by introducing central management and control of network reconfigurations [8,9]. These solutions mainly focus on channeling programmable sensor devices. These ideas have revolutionized the reconfigurations of WSNs, and extensive industrial- and academic-level ideological experiments have shifted towards this. Central management and reconfiguration control of the Software-Defined Networking (SDN) paradigm and OpenFlow currently comprise the most popular instance of SDN.

**Figure 1:**
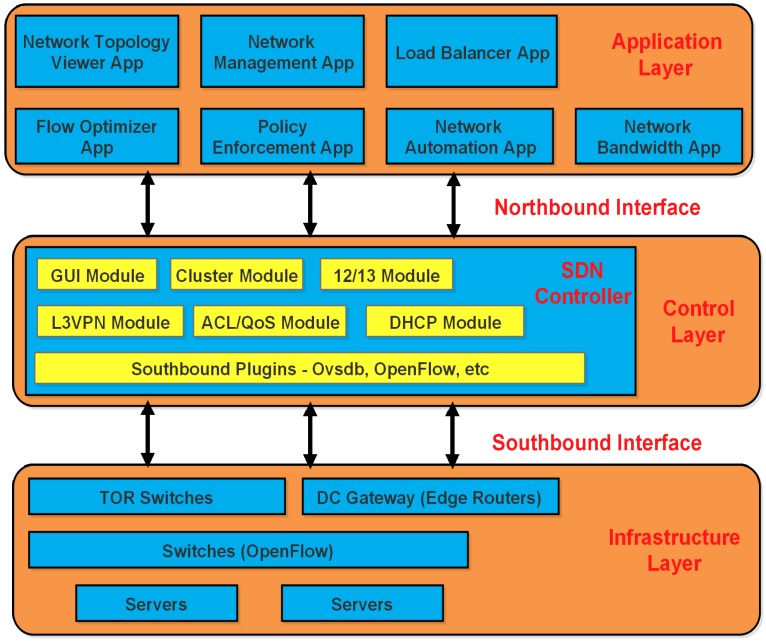
Architecture of Software-Defined Networking (SDN).

SDN has fundamentally evolved to bring the control plane to the central controller and impose the reconfigurable forwarding plane by installing centrally-programmable forwarding tables on network devices. Networking layer utilizes different modules like Access List (ACL) module for establishing forwarding tables. The basic centrally-oriented architecture of SDN is shown in Figure 1. Since practical solutions for central management of SDN penetration in wired and wireless enterprise networks have been developed, researchers have achieved a breakthrough in introducing similar SDN-based central management to WSNs. This provides the capability to shift complicated control logics like topology discovery, residual resource computations and path-selection to route the sensed reports from sensor nodes to the SDN central controller. Limited battery-oriented sensor nodes save a significant amount of energy by the replacing the control plane computation and only being responsible for the forwarding plane. Although nodes still have the burden of reconfiguring the forwarding tables on the iterative reconfiguration period, they save a significant amount of energy due to the absence of the control plane [8,9].

SDN-based iterative reconfiguration of WSNs is more suitable for energy-efficient cluster-based vertical reorganizations of network nodes into Forwarding Element Cluster-Heads (FECHs) and non-Forwarding Element Cluster-Heads (non-FECHs). The SDN central controller develops routing policies for horizontally-separated control from the data planes and equally redistributes the responsibilities of FECHs and non-FECHs. Therefore, the control plane optimizes the data-handling decisions, whereas the data plane forwards data according to the routing decisions of the control plane. Major clustering protocols emphasize developing the required standard energy-efficient features of iterative reconfigurations, including application-specific, rate adaptation and powering down approaches [10,11,12,13,14,15]. Rate adaptation results in the energy consumption reduction of networks by scaling the power consumption of a network element to the amount of traffic it carries. The power down approach conserves energy by switching off unused network elements, which operate either at the full rate or zero rate [4,16,17].

However, often, iterative reconfigurations degrade QoS for classical WSNs and introduce inertia into the system because application-specific sensing, rate adaptation and speed scaling create an overhead of intensive packet exchange between sensor nodes and the central controller at the BS. Hence, we may avoid them and thus not apply the most suitable computed routing configuration [18,19,20]. In this paper, we overcome the above shortcomings and go beyond the state-of-the-art protocols by defining two optimized energy-efficient routing protocols called SDN-based Application-aware Centralized adaptive Flow Iterative Reconfiguring (SACFIR) and the extended version of SDN-based Application-aware Main-value Centralized adaptive Flow Iterative Reconfiguring (SAMCFIR). In this paper, we propose highly adaptive and dynamic iterative reconfigurations for WSNs with the support of SDN-based centralized network management. This dynamic configuration periodicity is adjusted according to the network requirements of the remaining resources at the Top-Of-Rack (TOR) switches of infrastructure level, while upper layer resources are considered to be sufficient throughout the network period. Therefore, the SDN central control system offers complex computational decision making for continuous cycling development of global routing logics and reconfigures the network from time to time in order to maintain a low running cost for sensor nodes.

The rest of the paper is organized into the following sections: Section 2 presents some highlights of related state-of-the-art literature. Section 3 provides the technical details of the heterogeneous hybrid network model of SDN-WSN for the execution of the SACFIR and SAMCFIR protocols. In Section 4, we propose two routing protocols, SACFIR and SAMCFIR, and the analytical outputs of the application-specific reports of inter-networking communications. Section 5 presents the performance evolution of the SACFIR and SAMCFIR protocols. Finally, Section 6 concludes this paper’s contribution and highlights the future work.

## 2. Related Work

Extensive research has concentrated on the installation of reliable and affordable WSNs to identify core issues in traditional management systems. Industrial growth requires exclusive management systems to ensure the heterogeneity of network resources and a wide application spectrum. In this regard, significant research work is being conducted on the integration of the SDN management layer with the infrastructure layer of WSNs [21,22,23]. All these solutions tend to shift control logics to the centralized controller, and nodes obey their responsibilities of forwarding tasks. Existing research highlights many interesting developments that enable SDN-based networking capabilities for WSNs to enhance application-awareness, wide-area network management and the stability of network performance [24,25,26].

By reviewing the related literature in detail, we discuss the following contributions that are vital additions to this paper. The basic SDN-WSN integration architecture is given in [5] by proposing the idea of Software-Defined Networking for WIreless SEnsornetworks (SDN-WISE). SDN-WISE attempts to cover the theoretical layout and real-time deployment effort by making WSN nodes programmable during online configurations. However, this requires improvement for a better integration model, with WSNs supporting conventional cluster-based organization to allow practical installations to prosper.

In [27], SDN-WSN hybrid inter-networking is proposed while taking care of cluster organization; this integration is known as Software-Defined Clustered Sensor Networks (SDCSN). However, this scheme uses extensive resources by adding multiple BSs playing the role of CHs, and normal nodes only create clusters with these BSs. This architecture adds to the complexity of thenodes, as they act as sub-controllers. Moreover, it supports multiple controllers simultaneously. This causes extensive control message flooding during the setup phase and dissipates major energy resources of the WSNs. Adding to the versatility and flexibility of WSNs, the authors in [6] enabled a Network Management System (NMS) for central management of WSNs at the application level. The additional control layer of an SDN promises application-awareness, but these improvements show limited capacity at the theoretical level and many challenges for real-time execution.

Establishing a reliable intra-communication protocol between forwarding logics and central control logics is an additional challenge. This issue is addressed by the Sensor OpenFlow (SOF) [27,28] SDN-WSN environment. SOF develops flow table instructions generated at the controller to enable physical-layer forwarding tables at the sensor devices. These ideas are key technical contributions and provide intelligent components for advancements in this area. However, for real-time implementations, these ideas need actual prototypes that can offer significant participation. The soft-WSN architecture is proposed in [12]. The overall research proposes energy efficiency and network load-balancing in the hybrid network of the SDN-based Internet of Things (IoT) integration. Network configurations start from the central controller by application layer-defined decision making to ensure the utilization of limited network resources. However, these configurations are static and are not iterative in nature. These shortcomings imply the possibility to enhance the network performance. Another SDN-WSN framework called TinySDN based on Tiny-OS is proposed in [29], which contains multiple SDN-enabled sensor nodes guided by multiple SDN controllers. TinySDN based on Tiny-OS offers in-band control, which causes unacceptable latency and extensive energy consumption due to the lack of iterative clustering efficiency. One of the research surveys reports this system to be vulnerable to security collapse.

A similar central management-oriented software-defined sensor networking system was introduced in [30,31], mainly based on the TinyOS platform. This contribution is not suitable for dense WSNs, as it is designed for quick measurements for very short distances and less crowded SDN-WSNs environments. The Software-Defined Wireless Network (SDWN) is another considerable solution proposed in [32]. The SDWN extended prototype shows functionality for static and mobile sensor nodes. Energy consumption in mobile sensor nodes is drastic, and the duty cycling technique of SDWN enables some energy savings with the help of advanced data aggregation. In [16,17], centralized routing protocols for conventional WSNs include Application-aware Threshold-based Centralized Energy-Efficient Clustering (ATCEEC) and Multi-hop Centralized Energy-Efficient Clustering (MCEEC) protocols, which display energy efficiency and load-balancing for heterogeneous WSNs. Both protocols show considerable improvement, but still lack a real-time management system to deal with the practical implementations. Moreover, these protocols have static iterations for re-clustering, which continuously adds to the burden of the lower energy nodes.

In order to address the aforementioned issues, this paper proposes two SDN-based centrally-managed routing protocols for WSNs called SDN-based Application-aware Centralized adaptive Flow Iterative Reconfiguring (SACFIR) and the extended version of SDN-based Application-aware Main-value Centralized adaptive Flow Iterative Reconfiguring (SAMCFIR). SACFIR and SAMCFIR entail significant improvements. The comparison of the characteristics of the proposed models with existing state-of-the-art solutions is given in Table 1. The technical details of our proposed protocols are given in Section 3 and Section 6.

## 3. Heterogeneous Network Model of the Proposed Models

We extend the SDN-WISE architecture to establish SDN-enabled Iteratively Reconfigurable WSNs (SDN-IRWSNs) in order to execute our proposed routing protocols of SACFIR and SAMCFIR. The hybrid architecture of SDN-IRWSN is deigned to achieve the following essential objectives: (i) adaptive iterative reconfigurations allocate transmission functions to reprogrammable sensor nodes according to their residual resources to prolong their presence in the network; (ii) online reconfigurations are utilized to monitor the network environment through the complex computational assistance of the SDN central controller, which generates simple flow tables for forwarding sensor devices; (iii) to design programmable sensor nodes that are capable of sensing multiple applications simultaneously.

### 3.1. SDN-Enabled Iteratively Reconfigurable WSNs of the Proposed Models

**Figure 2:**
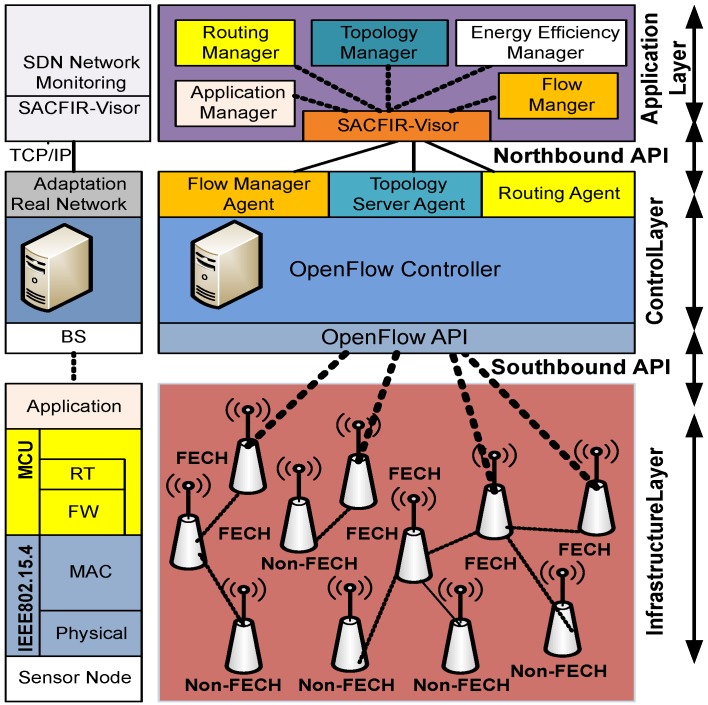
Heterogeneous hybrid architecture of SDN-enabled Iteratively Reconfigurable WSNs (SDN-IRWSNs).

The iterative architecture of SDN-IRWSNs consists of OpenFlow-enabled sensor nodes deployed at the infrastructure layer, while these sensors are connected to the SDN central controller, which resides at the Base Station (BS). The number of sensor nodes varies according to the required applications, and similarly, SDN-IRWSNs can be extended to utilize multiple SDN controllers. The basic architecture of SDN-IRWSNs is shown in Figure 2. The left side of Figure 2 indicates the protocol stack, while the right side indicates the SDN-IRWSN architecture’s layers and associated components. The infrastructure layer consists of sensor nodes, and these planes utilize the protocol stack of forwarding logics, while each sensor consists of multiple sensing components of environmental parameters. These sensor nodes have a Micro Controller Unit (MCU) with limited computational capacity powered by the Power Unit (PU) and Sensing Unit (SU). All the attached components are powered by PU to operate their circuitry. These sensor nodes include IEEE 802.15.4 transceivers for wireless communication with the central controller at BS. Forwarding (FW) layers deal with packet forwarding activities by adjusting forwarding tables for information received through IEEE 802.15.4 transceivers containing Receiver Transmitter (RT). These forwarding tables are dictated by control layer components that include single or multiple SDN controllers according to network scalability.

Similarly, at the protocol stack level, the Inter-Networking Processing (INP) protocol guides the path selection of the forwarding mechanism. Inter-Networking Processing (INP) is responsible for the path selection process, and routing decisions implemented on the SDN controller are executed here to define the routing tables. In this paper, we propose two routing policies, SACFIR and SAMCFIR, which are implemented at the INP level. In order to create an efficient forwarding table at the infrastructure layer, the SDN controller hands over some key network statics to the application layer to make the routing decisions, and this key information is obtained through the topology discovery layer. The control layer sends existing routing, flow-table and topology information to the application layer by SACFIR-Visor. SACFIR-Visor supervises the collected information sent to the application layer APIs and waits for feedback. The application layer APIs are programmable by the network administrator to respond according to the network conditions. Then, SACFIR-Visor ensures that the SDN controller implements the received response.

### 3.2. Network Topology for the Proposed Protocols

The network topology for the proposed model consists of the central controller residing at the BS, placed at the boundary of the network region, and a limited number of sensor nodes being scattered in the network area. Initially, the whole network region is assumed to be a two-dimensional rectangular area with A to B limits at the *x*-axis and C to D limits at the *y*-axis. Therefore, network area [A,B]×[C,D] is divided into *i* clusters along the *x*-axis and *j* along the *y*-axis. Every cluster position $\left(x_{i},y_{j}\right)$ represents an independent sub-area. The central controller at the BS can compute the height of the surface, $f(x_{i},y_{j})$, and the Average Surface Height (ASH) of all clusters is approximately equal to the height of the whole network, which can be calculated as:

(1) $$ASH=\frac{f\left(x_{o},y_{o}\right)+\ldots +f\left(x_{1},y_{1}\right)+\ldots +f\left(x_{i-1},y_{j-1}\right)}{I\times J}$$

Approximation sigma notation can be used to generalize this scalability:

(2) $$ASH\approx \frac{1}{IJ}\sum_{i=0}^{I-1}\sum_{j=0}^{J-1}f\left(x_{i},y_{j}\right)$$

where the total length of *i* and *j* can be defined as I=B−A and J=D−C. From this, the ASH scalability approximation can be derived as:

(3) $$ASH\approx \frac{1}{\left[B-A][D-C\right]}\sum_{i=0}^{I-1}\sum_{J=0}^{J-1}f\left(x_{i},y_{j}\right)▵x▵y$$

where ▵x and ▵y is the variation at *i* and *j*, respectively, and special notation for the limit of the double sum can be defined as:

(4) $$ASH\approx \lim_{IJ\to \infty }\sum_{i=0}^{I-1}\sum_{j=0}^{J-1}f\left(x_{i},y_{j}\right)▵x▵y$$

In this way, double integration optimization can be derived from the above expressions:

(5) $$\lim_{IJ\to \infty }\sum_{i=0}^{I-1}\sum_{j=0}^{J-1}f\left(x_{i},y_{j}\right)▵x▵y=\int_{A}^{B}\int_{C}^{D}f\left(x_{i},y_{j}\right)dA$$

(6) $$ASH=\lim_{IJ\to \infty }\int_{A}^{B}\int_{C}^{D}f\left(x_{i},y_{j}\right)dA$$

**Figure 3:**
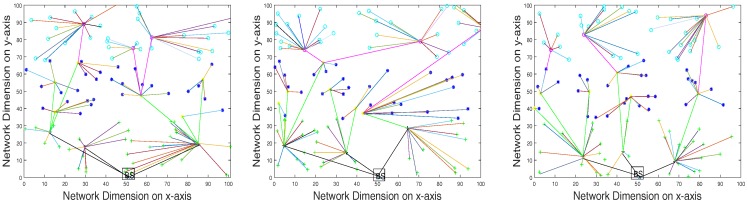
Iterative clustering topology in the case of the SDN-based Application-aware Centralized adaptive Iterative Flow Reconfiguring (SACFIR) routing protocol.

**Figure 4:**
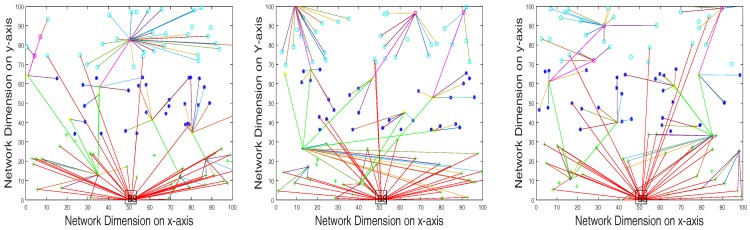
Iterative clustering topology in the case of the SAMCFIR routing protocol.

**Figure 5:**
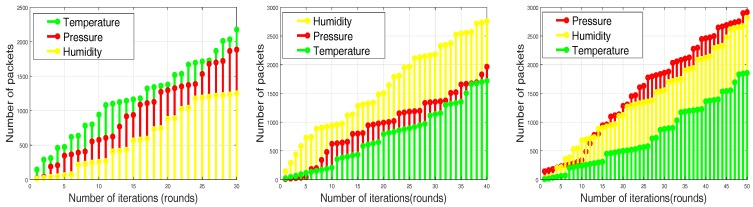
Application-sensitive environmental reporting of the SACFIR routing protocol.

**Figure 6:**
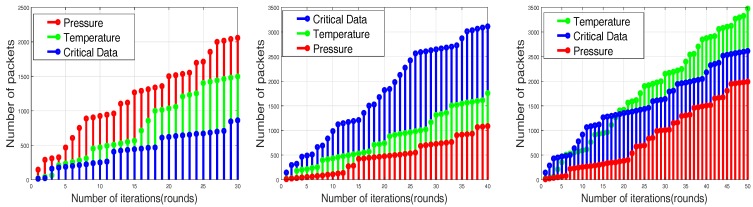
Application-sensitive environmental reporting of the SAMCFIR routing protocol.

Based on initial energy resources, sensor nodes are divided into three categories called normal nodes, advance nodes and super nodes, which contain different energy levels in ascending order, respectively. The central controller monitors the sensor layer-wise distribution according to energy level resources. The distribution and the iterative clustering of the sensor nodes are shown in Figure 3 and Figure 4. Multi-application sensor nodes scattered in the case of the SACFIR protocol are able to report environmental parameters like temperature, humidity and pressure. This application-awareness of multiple sensing tasks of the SACFIR model is shown in Figure 5. This figure shows the application-awareness of the proposed model by sensing temperature, pressure and humidity. More importantly, the SACFIR model can adjust the information index preference decided by the application layer. In the case of SAMCFIR, the central controller adjusts the application sensitivity for monitoring of the temperature and pressure according to a regular pattern. To deal with emergency situations, this model considers this as critical data and directly transmits the data to the central controller. These iterative application-aware sensing outputs are shown in Figure 6. Furthermore, SAMCFIR has the flexibility to adjust the sampling rate of the sensing environment and can collect information according to the required application.

### 3.3. Iterative Solver with Min-Cost Optimization

Iterative reconfigurations are required to save energy resources because sensor nodes have limited battery capacity and tend to leave the network operations once their batteries have drained out. Hence, the key is to rotate the operational responsibilities equally, so that the SDN central controller monitors the resources carefully and develops an intelligent iterative reconfiguration mechanism distributed along time periods *t*, where *t* = 1, 2, …, *T* with T>1. One time iteration length can be decided by the network administrator according to the remaining network resources, and the SDN central controller can reprogram sensor nodes dynamically on the network operation. This multiple time period network reconfiguration mechanism is defined as a 0–1 integer programming model. In the following, we concentrate on developing a realistic mechanism to achieve this performance optimization task.

The proposed protocols develop the cluster-based organization of sensor nodes and FECHs effectively communicate with the central controller of SDN. Therefore, an undirected graph $G=(C,R,rE_{C})$ represents the clustering network model of WSNs at the infrastructure layer; where *C* defines the set of FECHs decided by the SDN controller, *R* represents the existing links r=i,j, configuring the available FECHs i,jϵC, and $rE_{C}$ is the representation of residual energy resources. Every single link rϵR occupies the traffic capacity of $\alpha_{r}$ and has a cost of $\beta_{r}$; this as a whole defines the maximum amount of the flow roundtable for the current configuration settings, while the system has to bear the communication cost paid per unit of routed flow, respectively.

If a sensor node has a sensed value, it initializes a unicast demand of uϵU and a link with destination pair $\left(S_{u},D_{u}\right)\epsilon C^{2}$. The set *U* represents the active demands on this current network configuration and utilizes specific resources to route reports through the network. The SDN central controller needs to provide the most economical pathway to solve an evolving instance of the Minimum Cost Function (MCF) problem, which can be formulated at a given time as the linear program (1)–(3); where real variables $(X_{p})p\epsilon P$, $(Y_{r})r\epsilon R$ and $\left(Z_{e}\right)e\epsilon rE_{C}$ represent the path, link and residual energy utilization and take values in the range [0, 1]. The minimum cost function can then be expressed using the above-defined notations:

(7) $$Min-cost^{OPT}\left(X_{p},Y_{r},rE_{e}\right)=min\sum_{t=1}^{T}\sum_{r\epsilon R}Y_{r}\beta_{r}$$

(8) $$s.t.\sum_{t=1}^{T}\sum_{p\epsilon P_{u}}X_{p}=1$$

(9) $$s.t.\sum_{t=1}^{T}\sum_{e\epsilon rE_{u}}Z_{e}=1$$

(10) $$\sum_{t=1}^{T}\sum_{u\epsilon U}\sum_{p\epsilon P_{r}:u\epsilon P_{r}}X_{p}\Gamma_{u}\leq Y_{r}\alpha_{r}$$

The objective function (1) models the overall price paid for using the network links, and the constraints (2) ensure that the entire demand $\Gamma_{u}$ is routed through a set of paths with routing splits $X_{p}\Gamma_{u}$. Similarly, the constraints (3) are the residual energy constraints, and (4) are the link capacity constraints.

## 4. Proposed Model of SACFIR and SAMCFIR

In this section, we propose two routing protocols called SDN-based Application-aware Centralized adaptive Iterative Flow Reconfiguring (SACFIR) and its extended version, SAMCFIR. The proposed models are designed for centralized intelligent path selection for OpenFlow-enabled sensor nodes assisted by the SDN central controller residing at BS. The proposed model’s route computation at the SDN controller is periodic. This enables resource-awareness and application-sensitivity, allowing us to obtain feasible flow reconfiguration solutions. Flow reconfigurations take time and cause small disturbances that affect the QoS. The proposed protocols compute a feasible solution in every iteration, but the optimal path selection solution is reinstalled every 10th round to avoid redundant reconfiguration operational cost. The SDN controller sets the computational formula of the default reconfiguration threshold for the current period, which is calculated as:

(11) $$\begin{array}{c} RT_{q}=\left\{\begin{array}{cc} \frac{T_{v}}{\left(1-T_{v}\times (qmod\frac{1}{T_{v}}\right)}, & \mathrm{if}\;rE>0\\ 0, & \mathrm{otherwise} \end{array}\right. \end{array}$$

where $RT_{q}$ is the threshold value, $T_{v}$ is the desired percentage of the threshold value, with the default value of $T_{v}=0.1$, while qis the current period and rE>0 indicates the residual energy of alive nodes. This method achieves the major objective of the proposed protocols, which is to implement re-installation of the configurations with minimum cost by holding back the re-computed paths for a certain period. However, in certain situations when networks face a drastic performance decline and if the current period $Min-cost_{q}^{OPT}$ is much lower than previous period $Min-cost_{q-1}^{OPT}$, then the proposed models reset the current period value q=9. In this case, the SDN central controller considers the current iteration as the 10th period and performs an abrupt reconfiguration execution. The periodic route computation of the SDN controller executes the SACFIR and SAMCFIR routing algorithms, in which every round consists of three phases called the Network Topology Management Phase (NTMP), the Network Settling Phase (NSP) and the Network Forwarding Phase (NFP). The SACFIR and SAMCFIR routing algorithms share a similar procedure to NTMP and NSP, while the major differences are seen with respect to NFS. The details of the next three sub-sections provide the technical depth of the proposed models.

### 4.1. Network Topology Management Phase of SACFIR and SAMCFIR

Both proposed models adopt the topology manager functionality to maintain the global view of current network nodes at the central controller to compute flow configurations for the available resources. The SDN controller requires periodic updates about the network state to establish an efficient and reliable topology discovery mechanism, which is critical for the accuracy of every SDN-enabled system. Existing OpenFlow-enabled sensor devices do not support any dedicated functionality for topology discovery, which is why it is the sole responsibility of the controller to implement this service. The most widely-implemented SDN controller platforms are derived from the topology discovery of the conventional controllers POXand NOX [33,34]. Initially, this mechanism was known as the OpenFlow Discovery Protocol (OFDP), and, furthermore, OFDP leveraged the Link Layer Discovery Protocol (LLDP) [35]. This makes the exchange of LLDP the de facto SDN topology discovery standard, and generically, we refer to it as Topology Discovery (TD) packets.

In the case of our proposed models, the SDN controller iteratively broadcasts TD packets on the OpenFlow southbound channel simultaneously with existing forwarding instructions. These TD packets envelop basic information like the identity of the corresponding SDN controller in the case of multiple SDN controllers available at BS, residual energy resources and the distance range between the sensor node and the serving SDN controller. In static networking environments, all sensor nodes initially share neighborhood distance information, the RSSI level among neighbors, the density and their energy level at the Tier 1 level. Distance remains constant, while density and residual energy resources require the sharing of regular updates. In contrast, mobility scenarios require continuous sharing of the current distance to BS and the exponential increment or decrement of distance in the neighborhood. Because each sensor node periodically generates a TD packet containing its current list of neighbors and transmits it to BS, the SDN controller can notify the sensor node to clear its pervious list and recompute it. A higher rate of continuous generation of these TD packets affects the performance of the network with limited resources. As WSNs can afford small-scale topology information exchange due to the shortage of energy resources, the execution of SACFIR and SAMCFIR requires the overhead of the frequent generation of TD packets to be minimized. In default settings, the interval size of 5 s is considered to be the TD packet interval [34]. In the case of the proposed models, the SACFIR-Visor undertakes topology management according to the application-sensitivity and residual energy resources of active sensor nodes. Therefore, it can increase the TD packet interval value to avoid the overhead of extensive topology updates. The SACFIR and SAMCFIR routing protocols enhance the settling phase management because they have an efficient network topology from the viewpoint of the SACFIR-Visor.

### 4.2. Network Settling Phase of SACFIR and SAMCFIR

In this phase, the SDN central controller primarily computes the routing responsibilities and organizes the cluster-based reconfiguration schemes for WSNs nodes. The proposed protocols, SACFIR and SAMCFIR, and the utilized SDN central controller contain unlimited energy resources and higher computational capabilities to perform flexible network monitoring in every period and effectively develop a re-clustering configuration every 10th period.

During NTMP, the SDN controller receives updates from heterogeneous WSNs about their initial energy and distance from BS. During NSP, the SDN central controller maintains the global view of network energy resources and computes the average residual energy of the scattered sensor nodes by the linear equation of:

(12) $$\bar{rE_{i}\left(q\right)}=\frac{1}{N}\sum_{i=1}^{N}rE_{i}\left(q\right)$$

$\bar{rE_{i}\left(q\right)}$ is the average energy, and $rE_{i}\left(q\right)$ is the residual energy of sensor nodes.

BS compares the residual energy of each node (1≤i≥N) to their corresponding region’s average energy. Nodes with higher energy or average energy $\left(E_{i}\geq AverageEnergy\right)$ have a better chance of being selected as the Expected FECHs (EFECHs). However, the controller needs to consider other factors of distance and the centrality of nodes within the neighborhood. The minimum distance between ECHs and BS is calculated by equation:

(13) $$Min-dist_{toBS}{=}^{\sqrt{{\left(X-xj\right)}^{2}+{\left(Y-yj\right)}^{2}}}$$

Min−disttoBS is the minimum distance between BS and the *j*-th ECH. X and Y are the location coordinates of BS. Similarly, xj and yj are the location coordinates of the *j*-th ECH in the whole network region. The SDN controller has to select the FECHs with the maximum value of centrality, which is referred to as point centrality in graph theory [24]. Here, we consider nodes with different points, and nodes with a higher degree have better centrality and become the ideal nodes with which cluster member nodes link. The Clustering Centrality (CC) of an EFECH is the average ratio by which a source Sneighboring non-FECH needs to pass through a specific node EFECH to reach destination (D), which can be intermediate FECHs or BS, via the shortest path in the network. Therefore, the CC of a specific EFECH is given by:

(14) $$CC_{EFECH}=\sum_{S\neq EFECH\neq D}\frac{{}_{SD}\left(EFECH\right)}{{}_{SD}}$$

The ${}_{SD}$ is the total number of shortest paths from the source to destination, and ${}_{SD}\left(EFECH\right)$ is the number of shortest paths between S and D that pass through EFECHs. Clearly, a node with higher CC has greater influence in its network, and thus, it is prone to becoming FECH. In a dense network environment, the process of $CC_{EFECH}$ can nominate too many FECHs; in this situation, the SDN central controller selects AliveNodes×P FECHs with maximum residual energy and $CC_{EFECH}$.

BS announces these nodes’ FECHs by setting up their type and reconfigures the new configurations throughout the network. Every further FECH starts clustering by advertising the cluster membership request in the neighborhood. In the case of non-FECHs nodes, the SDN controller leaves them alone and does not exchange any further hello messages in order to save energy resources, which is why FECHs are responsible for carrying out the membership process. Furthermore, non-FECHs select the optimal FECHs based on the criteria of RSSI and minimum distance.

FECHs’ selection by the proposed algorithms is simple so as to avoid algorithm complexity, but the major task is to optimize the performance of reconfigurations according to the residual energy resources and workload shared by FECHs. In this regard, SDN controllers continue analytical operations to redistribute the responsibilities evenly in the next reconfiguration period. Sensing and processing cost very little energy, which is why the SDN controller ignores this factor and concentrates on communication load to optimize the reconfigurations.

As *N* sensor nodes are clustered into FECHs and non-FECHs, the major energy cost is the sum of intra-cluster communications and inter-cluster communications. Initially, non-FECHs generate *f* reports with $f\epsilon \left[1,F\right]$, and we assume that this is the identifier of the application type supported by the node. A non-FECH *i* supporting the *f*-type application transmits corresponding information to the cluster’s FECH, denoted by $\Gamma_{if}$, and *j* FECHs receive all member data denoted by $\Psi_{jf}$. For all FECH data received at a single cluster, FECHs is denoted by $\sum_{j=1}^{N}\Psi_{jf}=1$ for all *f*. The transportation communication cost from *i* non-FECH to *j* FECHs is represented by $Z_{ij}$. Similarly, communication cost between FECH and *s* BS is denoted as $Z_{js}$. Our basic assumption enforces the idea that the SDN central controller understands the network topology and remaining resources of all sensor nodes, so it can predict the analytical consumption of energy resources and can compute the overall communication cost of an existing routing configuration. Let $\varrho_{f}$ be a binary variable taking values 0 and 1 to indicate, respectively, whether non-FECHs have generated a value or not. The overall system of intra-cluster cost from report generation to transmission towards FECHs can be calculated as:

(15) $$Intra-C-Cost\sum_{f=1}^{F}\sum_{i=1}^{N}\sum_{j=1}^{N}Z_{ij}\Gamma_{if}\Psi_{jf}\varrho_{f}$$

Similarly, inter-cluster communication cost is calculated by:

(16) $$Inter-C-Cost\sum_{f=1}^{F}\sum_{j=1}^{N}l\varrho_{f}Z_{js}\Psi_{jf}$$

where *l* is the amount of aggregated information transmitted to BS. From the above two equations, the SDN controller can calculate the total communications cost.

(17) $$Total-C-CostIntra-C_{C}ost+Inter-C_{C}ost$$

(18) $$Total-C-Cost\sum_{f=1}^{F}\sum_{i=1}^{N}\sum_{j=1}^{N}Z_{ij}\Gamma_{if}\Psi_{jf}\varrho_{f}+\sum_{f=1}^{F}\sum_{j=1}^{N}l\varrho_{f}Z_{js}\Psi_{jf}$$

(19) $$Total-C-Cost\sum_{f=1}^{F}\sum_{j=1}^{N}\Psi_{jf}\left(l\varrho_{f}Z_{js}+\sum_{j=i}^{N}Z_{ij}\Gamma_{if}\right)$$

where the right side of the equation is constant for any node *i* to transmit *f* reports through *j* FECHs, thus it can be replaced by constant:

(20) $$Total-C-Cost\sum_{f=1}^{F}\sum_{j=1}^{N}\varsigma_{jf}\Psi_{jf}$$

Hence, the major performance goal of our proposed models is to deal with the cost function minimization. The basic analytical task for the SDN controller is to calculate the possible minimum cost to achieve energy efficient reconfigurations. This minimum cost can be represented by the following inequalities:

(21) $$\begin{array}{c} Min-Total-C-Cost\sum_{f=1}^{F}\sum_{j=1}^{N}\varsigma_{jf}\Psi_{jf} \end{array}$$

(22) $$\begin{array}{c} subject-to\sum_{j=1}^{N}\Psi_{jf}=1,\forall f\epsilon \left[1,0\right] \end{array}$$

(23) $$\begin{array}{c} \Psi_{jf}=0,1\forall j\epsilon \left[1,N\right] \end{array}$$

The above problem description has the property of a binary integer program and it holds class NP-Hard complexity in general. For these problems, greedy algorithm executions are needed to experiment with all possible solutions by testing every combination. The SDN central controller has enough computational and energy resources to carry out complicated analytical operations. However, we have to consider the limitations of the capacity of real-time WSNs nodes at the infrastructure layer regarding sensing, processing and transmissions capabilities. At this particular instance, we focus on processing capabilities by certain quantitative values which is denoted by Fmax. Now SDN controller’s analytical operation will be more restricted;

(24) $$\begin{array}{c} Min-Total-C-Cost\sum_{f=1}^{F}\sum_{j=1}^{N}\varsigma_{jf}\Psi_{jf} \end{array}$$

(25) $$\begin{array}{c} subject-to\sum_{f=1}^{F}\Psi_{jf}\leq Fmax\forall j\epsilon \left[1,N\right] \end{array}$$

(26) $$\begin{array}{c} \sum_{j=1}^{N}\Psi_{jf}=1,\forall f\epsilon \left[1,0\right] \end{array}$$

(27) $$\begin{array}{c} \Psi_{jf}=0,1\forall j\epsilon \left[1,N\right] \end{array}$$

Deployed sensor nodes at infrastructure layer face bandwidth limitation due to wireless domain and limited transceiver capabilities. Dense and large-area WSNs are bound to implement multi-hoping transmission that exacerbate bandwidth limitation issues. If the SDN controller sets a bandwidth threshold on the FECHs to carry out the transmission of its cluster nodes then we end up with the following problem formulation:

(28) $$\begin{array}{c} Min-Total-C-Cost\sum_{f=1}^{F}\sum_{j=1}^{N}\varsigma_{jf}\Psi_{jf} \end{array}$$

(29) $$\begin{array}{c} subject\alpha_{l}\geq \beta_{l}\forall l\epsilon \left[1,N\right] \end{array}$$

(30) $$\begin{array}{c} \sum_{j=1}^{N}\Psi_{jf}=1,\forall f\epsilon \left[1,0\right] \end{array}$$

(31) $$\begin{array}{c} \Psi_{jf}=0,1\forall j\epsilon \left[1,N\right] \end{array}$$

The basic cluster-formation algorithm of SACFIR and SAMCFIR by SDN controller is described in Algorithm 1.
**Algorithm 1:** SACFIR and SAMCFIR Reconfiguration Algorithm of FECHs
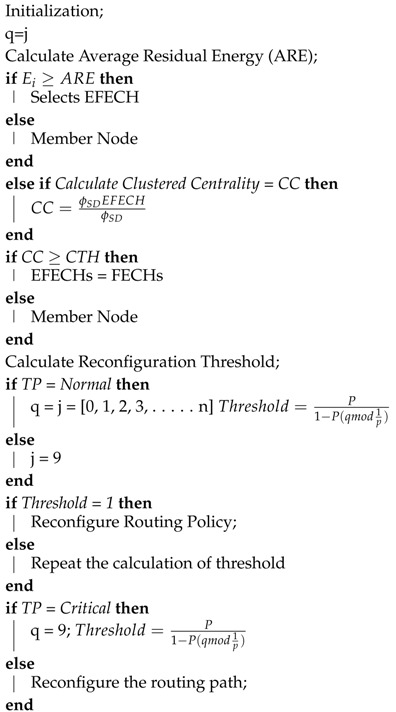


### 4.3. Network Forwarding Phase (NFP) of SACFIR and SAMCFIR

The real communication of network traffic transportation happens during NFP, when NSP has decided upon the reconfigurations of the updated forwarding rules for FECHs and non-FECHs. Initially, all nodes collect environmental reports by using multi-sensing utilities and MCU drives the digital reports from a continuous analog signal. Because sensor nodes are reprogrammable by SDN controller, we can reset their sensing preference according to the required applications. The SDN controller centrally sets the application characteristics during the initial hello packets. However, for routing, the analytical operation proposed model develops a threshold-based nodes sensitivity operation. A random number is generated and compared with the defined threshold preference which is decided by the network administrator for routing algorithm. The sensor nodes produce results according to the threshold values. The SACFIR routing algorithm is responsible for measuring three types of environmental parameters; temperature, pressure and humidity. Analytical operation of threshold based application-awareness is represented by equation:

(32) $$App-Thresh=\left\{\begin{array}{ccc} Temperature & If & Ran-Num<1stlevel\\ Pressure & If & 1stlevel<Ran-Num>2ndlevel\\ Humidity & If & Ran-Num>2ndlevel \end{array}\right.$$

Similarly, the SAMCFIR routing protocol is responsible for measuring temperature and pressure environmental attributes but it gives the flexibility to transmit critical data at sudden direct transmission without any significant delay. This is achieved by setting a critical transmission threshold, which can be calculated by the following equation:

(33) $$App-Thresh=\left\{\begin{array}{ccc} Critical & If & Ran-Num<1stlevel\\ Temperature & If & 1stlevel<Ran-Num>2ndlevel\\ Pressure & If & Ran-Num>2ndlevel \end{array}\right.$$

Application-specific transmission constitutes the major difference between SACFIR and SAMCFIR. Another major difference is the path selection difference in the transmission phase. SACFIR utilizes a unique multi-path inter-cluster communications for all types of environmental reports while SAMCFIR follows the same multi-path communication but also triggers the nodes for direct transmission when critical information is generated. All of these application-specific threshold priorities are reconfigured by the SDN controller during the settling phase. The network transmission phase can also be called the forwarding phase which is guided by the SDN controller logics at the control layer.

**Figure 7:**
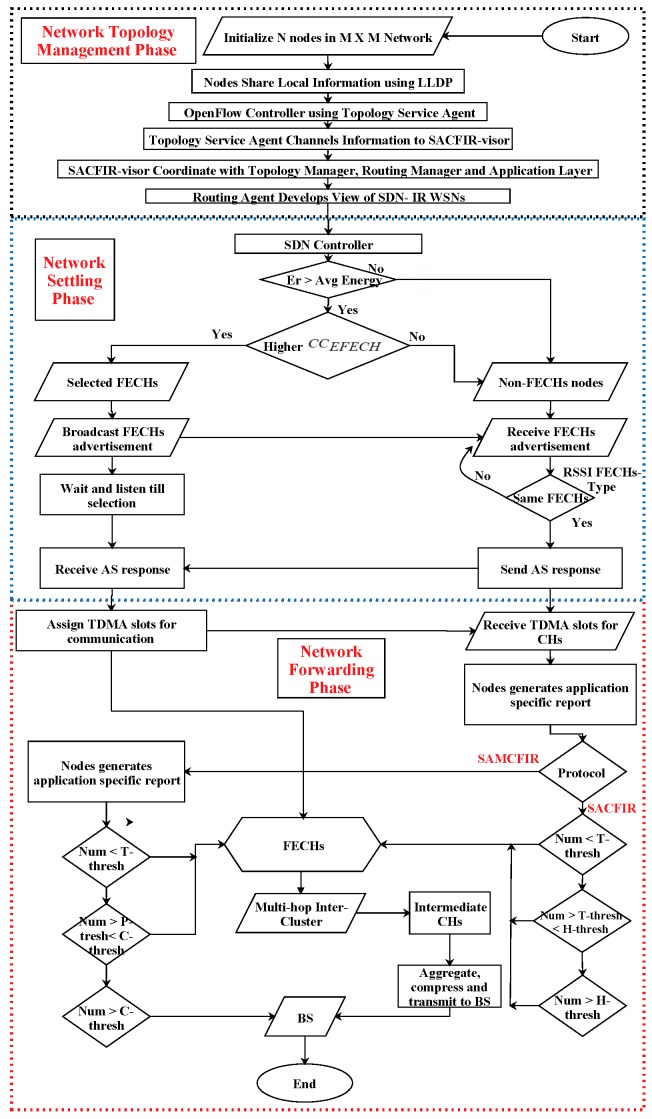
Flow chart proposed models.

The detailed flow-chart of the proposed models is shown in Figure 7.

#### Optimization in Multi-Hoping Communication

Optimization of the network throughput becomes crucial in the case of implementing the multi-hoping technique for limited resources of WSNs. The SDN controller develops graphical representation of the FECHs and computes energy and associated link capacities. This whole idea is extracted from the idea of multi-commodity flow.

Graphical representations of the selected FECHs are driven by $G_{FECHs}=\left(V_{FECHs},E_{FECHs}\right)$ in which M vertexes, $V_{FECHs}=1,2,\ldots ,M$, indicates FECHs and $E_{FECHs}$ edges(*i*, *j*) communicate FECHs within the transmission range.

Let us assume that there are *L* pairs of $FECHs_{V}\left(S^{l},D^{l}\right)$ for (*l* = 1, 2 …, *L*) which indicate the pair of source and destination $FECHs_{V}$ from the set of *L* commodities for which a transmission path is needed to be found on the graph $G_{FECHs}$. Every commodity has the following elements which must be be defined:

$\tau_{i,j}^{l}$ = Number of transmissions of *l* routed on $E_{FECHs}$ from *i* to *j*;

$T_{}^{l}$ = Total number of transmissions, transmitted on $V_{FECHs}\left(S^{l},D^{l}\right)$;

$\tau_{i,j}^{}=\sum_{l=1}^{L}\tau_{i,j}^{l}$ Total number of transmissions on communication link from *i*, *j*

So, every $FECHs_{V}$ has following communication responsibilities:

(34) $$X\left(FECH_{V}\right)=\left\{j\in V_{FECHs}|\left(CHs_{V},j\right)\in E_{FECHs}\right\}$$

(35) $$Y\left(FECH_{V}\right)=\left\{j\in V_{FECHs}|\left(FECHs_{V},j\right)\in E_{FECHs}\right\}$$

while, $X(FECH_{V})$ identify selected $FECHs_{V}$ that can be accessed by $FECH_{V}$, similarly $Y(FECH_{V})$ are $FECHs_{V}$ that can communicate directly with $FECH_{V}$ in the communication area. The enhancement of total deliverable traffic introduces a parameter ϕ that indicates total traffic fraction of $T_{}^{l}$ communicated within network. The optimum $\phi^{⋆}$ is the particular value of ϕ that satisfies the following objective function:

(36) $$Max\left\{\sum_{l=1}^{L}\phi \times T_{}^{l}\right\}$$

Further, conservation equations and non-negativity must be satisfied by all flows:

(37) $$\sum_{j\in X(FECH_{V})}\tau_{i,j}^{l}-\sum_{j\in Y(FECH_{V})}\tau_{i,j}^{l}=\left\{\begin{array}{cc} \phi \times T_{}^{l} & if\;S^{l}=FECH_{V}\\ -\phi \times T_{}^{l} & if\;D^{l}=FECH_{V}\\ 0 & otherwise \end{array}\right.$$

(38) $$\tau_{i,j}^{l}\geq 0\;for\;\left(i,j\right)\in E_{FECHs}$$

(39) ϕ∈[0,1]

From the above equation it is clear that positive transmission indicates source $FECH_{V}$, negative transmission represents destination $FECH_{V}$ and the zero value represents intermediate $FECH_{V}$.

Modeling interference to judge the capacity of the first cluster C of FECHs is initialized by adjacent $S_{i}^{l}$ to $FECH_{V}$

(40) $$C_{1}^{l}=\left\{\left(i,j\right),j\in V_{FECHs}\right\}$$

Similarly, for every node j A(i),cluster $C_{(}{i,j)}^{2}$, j contains every path that has one of its destinations in A(i) and has no relation with $C_{(}{i,j)}^{1}$:

(41) $$C_{(}{i,j)}^{2}=\left\{(j,k)|k\neq i\right\}$$

From above clusters, it is convenient for the SDN controller to develop a group of $G_{FECHs}$ generated by all intermediate nodes within two hops from the source *i*, which can be represented by:

(42) $$G_{CH_{i}}=\left\{C_{1}^{l}+\underset{j}{⋃}C_{(}{i,j)}^{2}\right\}$$

(43) $$\sum_{k=1}^{K}\sum_{\left(i,j\right)\in C_{i}^{b}}\tau_{i,j}^{l}+Max\left\{\sum_{k=1}^{K}\sum_{\left(i,j\right)\in C_{i}^{b}}\tau_{i,j}^{l}\right\}\leq F_{i}\forall G_{FECHs_{i}}$$

From the above outcomes, the SDN central controller is able to transfer the nonlinear constraint into a group of linear constraints and develops a linear model for our problem. In this way, during the forwarding phase, SDN controller contributes at the BS level and invests a lot of resources on the path selection computational function to manage WSNs nodes efficiently at the infrastructure level.

## 5. Simulation Results and Discussion

In this section, we critically analyze the achieved outcomes of the proposed model of SACFIR and SAMCFIR. The performance measuring parameters of network lifetime, stability, end-to-end delay and packet delivery ratio are considered most valuable to analyze. Extensive simulations of each experiment provide the average results of the proposed models in comparison with ATCEEC and MCEEC protocols. Conventional SDN controllers have flexibility issues in scalable wireless sensor networks, which is why recently-evolved solutions for SDN-WSNs have involved researchers proposing their own controllers according to the architecture and requirement of the sensor networks. A similar methodology has been adopted by this paper’s contribution. This paper presents the SDN-enabled Iterative Reconfigurable WSNs (SDN-IRWSNs) hybrid network architecture for the deployment of sensor nodes. We develop our own SDN controller to execute the Network Topology Management Phase (NTMP), Network Settling Phase (NSP) and Network Forwarding Phase (NFP) of proposed SACFIR and SAMFIR routing protocols.

In the general measurement process, we included specific network scenarios and omitted the other results due to space constraints. More specifically, the simulation scenarios included network areas of 100 m × 100 m, 300 m × 300 m and 450 m × 450 m, in which 100, 200, and 300 sensor nodes are dispersed respectively at the infrastructure layer. These sensor nodes are connected with SDN controller resides at BS, where BS is located at the top of the network as shown in Figure 3. Other important simulation parameters are given in Table 2.

### 5.1. Overall Network Operational Period and Stability Period

**Figure 8:**
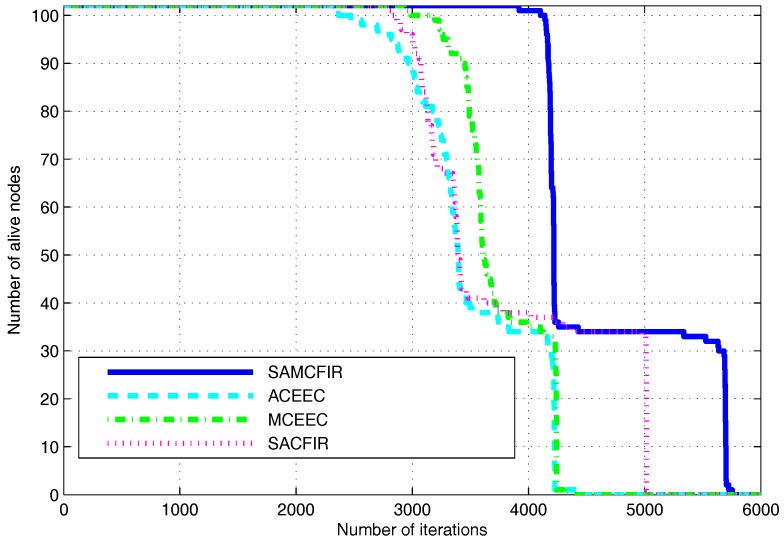
Energy Efficiency in terms of network lifetime in network of 100 m × 100 m with 100 nodes.

Figure 8, indicates the network lifetime and stability period of simulated protocols for first scenario experiment. Network lifetime depicts the time intervals between idealization and the expiration of all nodes, while stability period covers the time interval for which all nodes are functional (alive). In this case, SACFIR outperforms with 19%, which is 16% better network lifetime than ATCEEC and MCEEC respectively. We consider this improvement to be due to the SDN controller reconfigurations intelligence that develop the routing scheme for every period but are held back from implementing reconfigurations until the existing schemes produce acceptable forwarding. Similarly, SAMCFIR achieves 36%, which is a 32% network lifetime improvement on ATCEEC and MCEEC protocols. Becuase the forwarding phase of SAMCFIR limits the forwarding of similar information and waits until critical information arrives, it saves more energy and results in a better network lifetime. In the case of stability, SACFIR produces a 20% improvement on ATCEEC, while MCEEC still has a 2% better performer because MCEEC also utilizes the multi-hoping facility to reach BS. SAMCFIR develops better routing stability on both ATCEEC and MCEEC protocols with a margin of 64% and 41% respectively.

**Figure 9:**
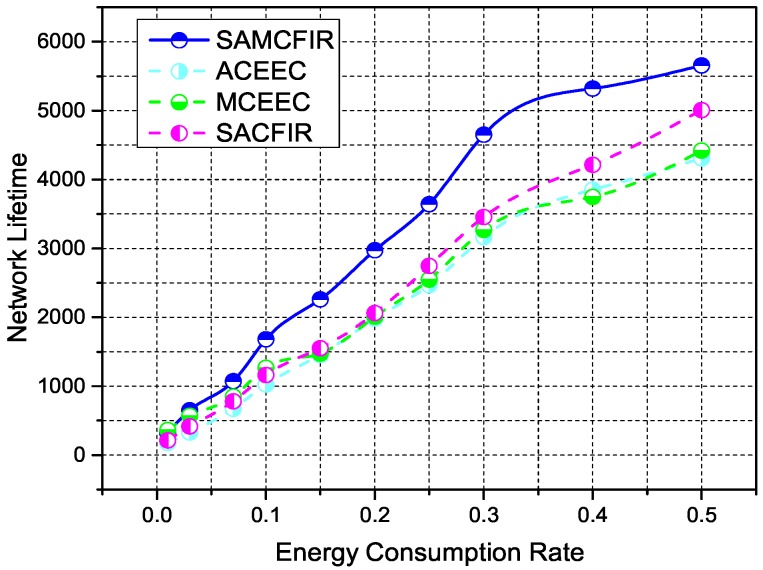
Energy consumption rate as network operation progress in network of 100 m × 100 m with 100 nodes.

Figure 9 shows the simulation results of average energy consumption ratios for ATCEEC, MCEEC, SACFIR and SAMCFIR protocols. Average energy consumption ratio means the amount of energy consumption per period of network iterative operation. In this case, the ATCEEC performs the worst as it has limited central route selection and carries the burden of single-hop forwarding. MCEEC has some improvement and the proposed model SACFIR and SAMFIR produce the optimal energy consumption utilization and extend the network lifetime to exceed the performance on existing protocols. The SDN central controller adds skills to the sensor nodes to perform reprogrammable configuration while understanding the remaining energy index. These features result in additional advantages of the proposed models to enhance an overall better network lifetime and stability period.

**Figure 10:**
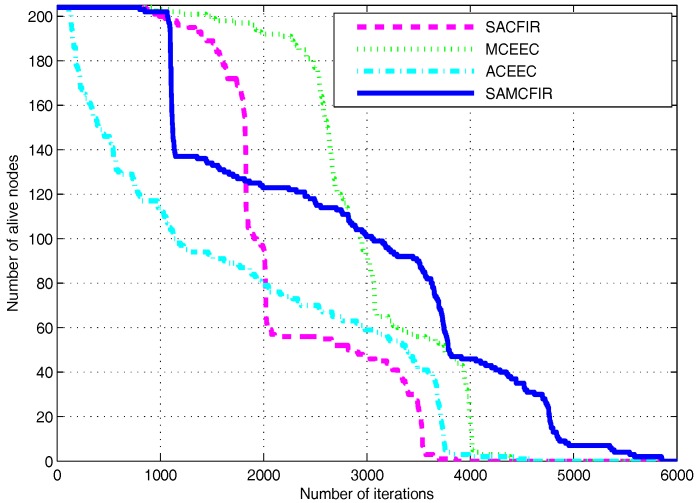
Energy Efficiency in terms of network lifetime in network of 300 m × 300 m with 200 nodes.

**Figure 11:**
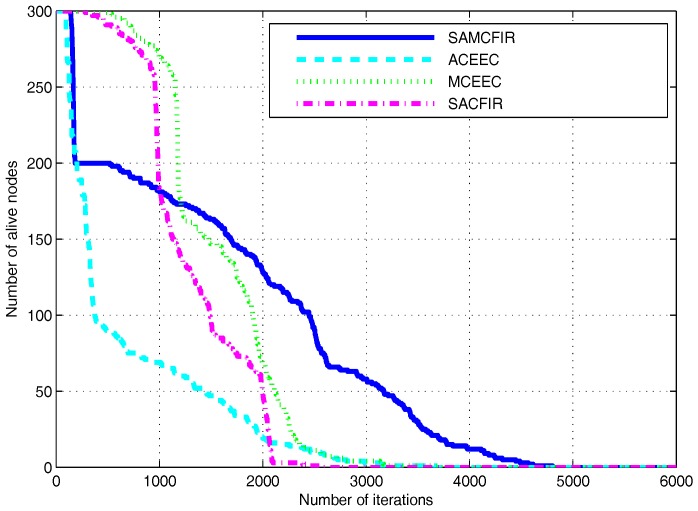
Energy Efficiency in terms of network lifetime in network of 450 m × 450 m with 300 nodes.

**Figure 12:**
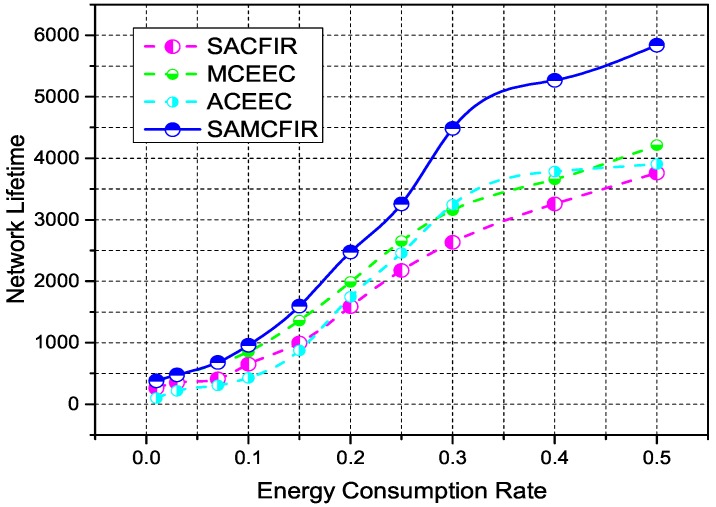
Energy consumption rate as network operation progress in network of 300 m × 300 m with 200 nodes.

**Figure 13:**
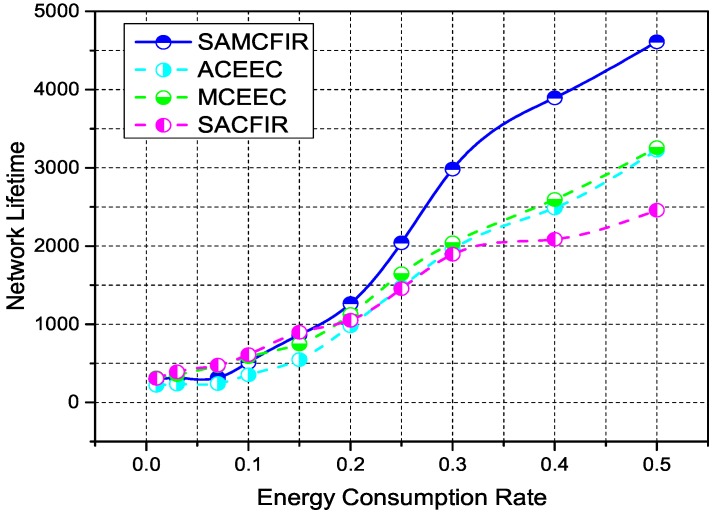
Energy consumption rate as network operation progress in network of 450 m × 450 m with 300 nodes.

Figure 10 and Figure 11 show the simulation outcomes regarding network lifetime and stability period for a network area of 300 m × 300 m and 450 m × 450 m, respectively. As a network area increases, the performance declination is more visible for all experimental protocols. However, the proposed models still show resistance and maintain an acceptable performance even in the most challenging and drastic network environments. Similarly, Figure 12 and Figure 13 represent the energy consumption ration for 300 m × 300 m and 450 m × 450 m respectively. The proposed models face drastic performance downfall but still show better resistance than ATCEEC and MCEEC.

### 5.2. Proposed Model Throughput, Packet Delivery Ratio, and End-To-End Delay

**Figure 14:**
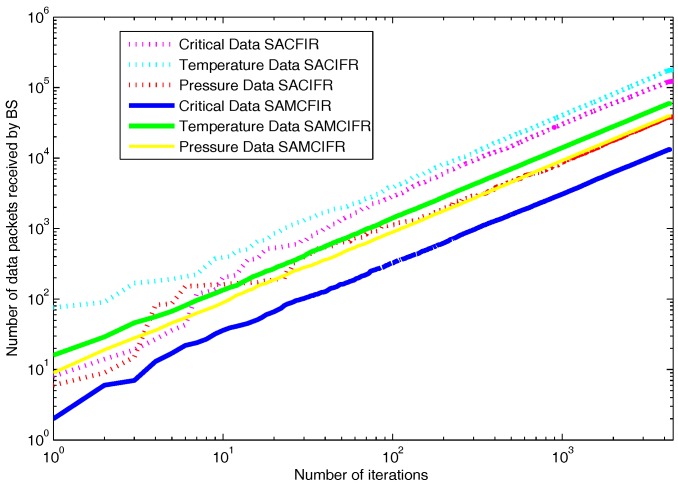
Multi-type data received at BS on network operation in a network of 100 m × 100 m with 100 nodes.

**Figure 15:**
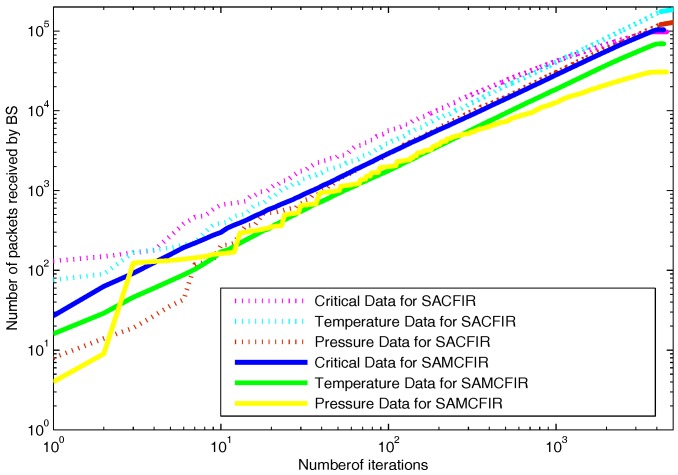
Multi-type data received at BS on network operation in network of 300 m × 300 m with 200 nodes.

**Figure 16:**
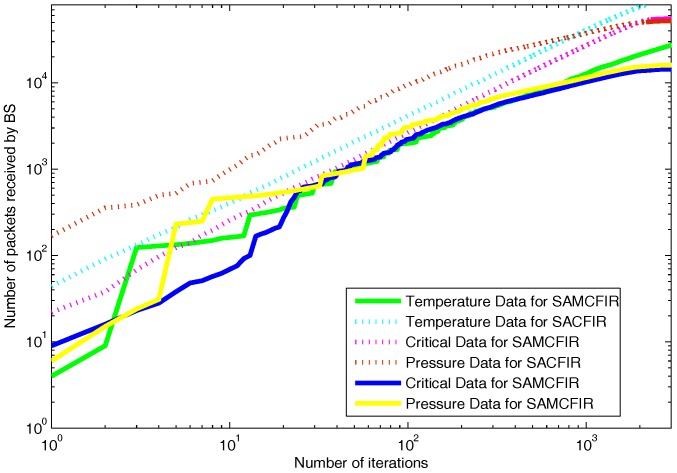
Multi-type data received at BS on network operation in network of 450 m × 450 m with 300 nodes.

As we mentioned above in detail, that proposed model routing patterns are centralized and driven by the SDN controller. Sensor nodes are capable of sensing multi-application environmental parameters. So the final reports contain the information about different sensed data as shown in Figure 14. Figure 14 depicts the results in a scenario of 100 m × 100 m, in which both SACFIR and SAMCFIR produce results according to threshold value defined by the central controller. Figure 15 and Figure 16 show the results for 300 m × 300 m and 450 m × 450 m networks respectively. As we can observe in every scenario, the amount of different types of information is different. This is because of the application preference reset of the SDN controller. Thus, reconfigurations bring flexibility to receive the required application data for a particular time interval from a single sensor node.

In conventional settings, the ideal assumptions define the optimistic transmission model. This optimistic model knowingly ignores signal distortion effects such as: interference, fading and signal attenuation. Furthermore, the control station unfolds the enveloped reports with 100% success without considering the sampling error rate. However, in the real network scenario, these challenges occur and need careful probabilistic measurement to calculate more actual efficiency. Current performance evolution in the presence of SDN controller management adopts a uniform random distribution model, which determines packet drop ($P_{d}$) probability. $P_{d}$ changes dynamically according to the network mobility pattern and distance between sensor nodes and BS. Link quality plays the tie breaker role and always needs to be high to compete with $P_{d}$ for better packet delivery. The deterministic value of $P_{d}$ is the following:

(44) $$P_{DSH}=\left\{\begin{array}{cc} 0, & if\;0\leq dist\leq 30\\ \left(\frac{1}{70}\right)\times \left(dist-30\right)) & if\;30\leq dist\leq 100\\ 1, & if\;dist>100 \end{array}\right.$$

$P_{DSH}$ is packet drop probability in single-hop inter-cluster communication conditions. $P_{d}$ in multi-hop inter cluster communications will be less drastic. Because FECHs uses short range intermediate FECHs, it can be expressed as:

(45) $$P_{DMH}=\left\{\begin{array}{cc} 0, & if\;0\leq dist\leq 30\\ \left(\frac{1}{95}\right)\times \left(dist-30\right)) & if\;30\leq dist\leq 100\\ 1, & if\;dist>100 \end{array}\right.$$

**Figure 17:**
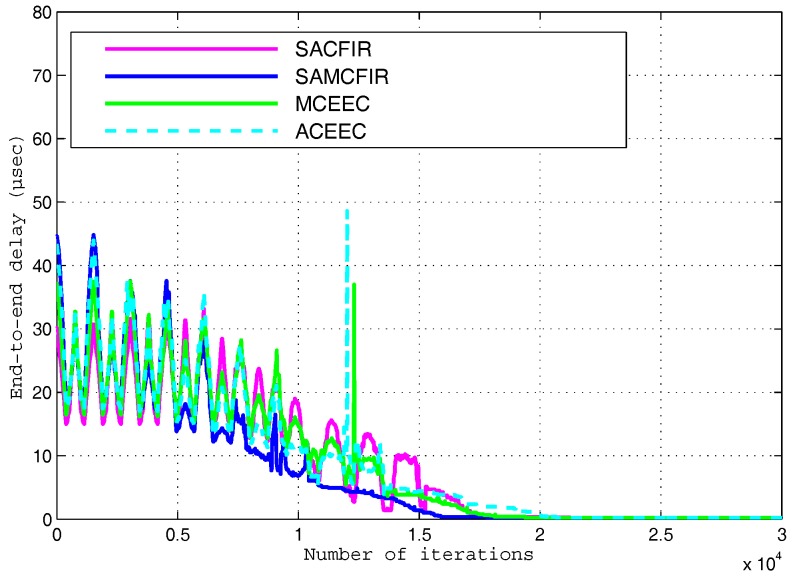
End-to-End delay experienced by execution of compared protocols.

**Figure 18:**
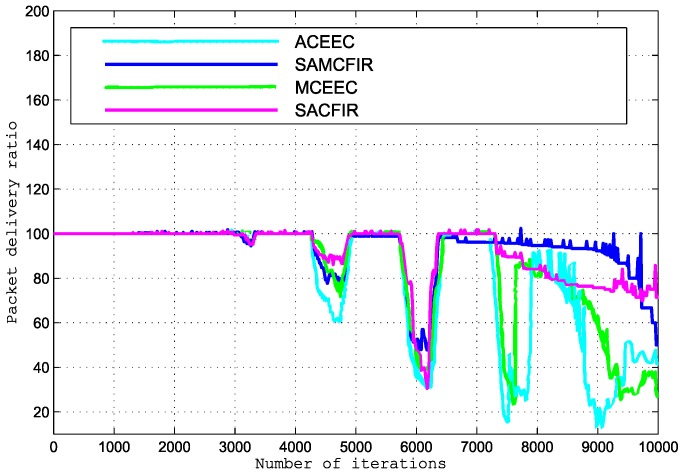
Packet delivery ratio experienced by execution of compared protocols.

In order to measure the packet drop ratio in detail within simulation environments, we increase initial energy level of all nodes to get better observations for longer period network operation. We also extend the network operational duration to 10,000 iterations to observe the behaviors of the protocols we are comparing. Figure 17 shows the encouraging results in which the proposed model is able to reduce the end-to-end delay. A higher rate of end-to-end delay in the ATCEEC and MCEEC is visible in these results. Figure 18 shows that the packet delivery ratio of SACFIR and SAMCFIR is much better than the other protocols of ATCEEC and MCEEC. The major reason behind this development suggests that the nodes start getting drained out. If there are fewer nodes that remain alive, this causes unwanted phenomena of higher $P_{DSH}$ as compared to multi-hop $P_{DMH}$. Our proposed routing protocols enabled the centralized SDN controller and reprogrammable sensor nodes which outperform the ATCEEC and MCEEC in all departments. In Figure 18, the MCEEC and ATCEEC experience drastic variation in last 4000 iterative periods of network operation. These unwanted performance fluctuations occur due to limitations of the heterogeneous network model for which ACEEC and MCEEC routing protocols are designed. Nodes scattered in heterogeneous network models of ATCEEC and MCEEC are divided into three categories and deployed into different areas according their energy order. These nodes have limitations of clustering process because nodes can only be clustered with their type of neighbor nodes. These limitations initially support ATCEEC and MCEEC executions but once nodes of distant regions start dying then the remaining nodes have difficultly to find CHs. Hence, the living nodes are restricted to limited region clustering formation that cause performance degradation in the latter part of the network operations. The proposed models show better resistance to such unwanted performance fluctuations because nodes deployed in the whole network region can take part in clustering with every available node with the assistance of the centralized SDN controller.

In the simulations given above, we have changed the number of nodes and size of network areas for different environments. The basic reason behind these variations in simulations setting is to analyze the performance in different network areas to observe the behavior of proposed protocols in different network density and scalability levels.

## 6. Conclusions

In this paper, we proposed two SDN-enabled centralized energy-efficient routing protocols called SDN-Based Application-aware Centralized adaptive Flow Iterative Reconfiguring (SACFIR) and SDN-Based Application-aware Main-value Centralized adaptive Flow Iterative Reconfiguring (SAMCFIR). Both proposed protocols are designed for an SDN-enabled Iteratively Reconfigurable WSNs (SDN-IRWSNs) network model to achieve central management with the help of an SDN controller. The sensor nodes deployed in the SDN-IRWSNs network arrangements have reprogrammable capabilities and support online reconfigurations during network operation. Sensor nodes utilize multiple sensing components to monitor multiple applications at the infrastructure layer while accepting dictation by the centralized control layer SDN controller to reset basic sensitivity and communication preferences. At the INP level, routing policies are generated at the application layer and implemented at the central controller to enforce forwarding mechanisms of sensor nodes. Our proposed routing protocols of SACFIR and SAMCFIR are implemented at the control layer, in which the SDN controller implements the proposed models to organize the network into clusters by selecting efficient FECHs. Furthermore, our proposed protocols offer advance dynamic reconfiguration capabilities which were lacking in existing centralized protocols such as ATCEEC and MCEEC. This dynamic reconfiguration ability of proposed models removes the overhead that might be caused by too many reconfigurations and reprogramming of sensor nodes, to achieve realistic multi-sensing capabilities. Extensive simulations result validate the better performance of our proposed models. In future work, we plan to implement the proposed models in a real-time network environment to achieve promising outcomes.

## Figures and Tables

**Table 1:** SDN-based sensor network and topology management architectures. WISE, WIreless SEnsor.

Management Architecture	Energy Efficiency	Load-Balancing	Scalability	Dynamic Reconfigurations	Resource Monitoring	Topology Management
SDN-WISE [5]	✓	✓	✓			
SDCSN [27]	✓			✓	✓	✓
Sensor OpenFlow [28]	✓	✓				
Soft-WSN [12]	✓		✓		✓	
TinySDN [29]	✓		✓			✓
TinySDM [30]	✓	✓	✓			✓
SDWN [32]	✓				✓	✓
ATCEEC [17]	✓		✓			
MCEEC [16]	✓		✓		✓	
SACFIR	✓	✓	✓	✓	✓	✓
SAMCFIR	✓	✓	✓	✓	✓	✓

**Table 2:** General simulation parameters.

Parameter	Value
1st Network size	100 m × 100 m
2nd Network size	300 m × 300 m
3rd Network size	450 m × 4500 m
Initial Energy	0.5 j
P	0.1 j
Data Aggregation Energy cost	50 pj/bit j
1st Network nodes	100
2nd Network nodes	200
3rd Network nodes	300
packet size	4000 bit
Transmitter Electronics (EelectTx)	50 nj/bit
Receiver Electronics (EelecRx)	50 nj/bit
Transmit amplifier (Eamp)	100 pj/bit/m2

## Abbreviations

The following abbreviations are used in this manuscript:

SDN	Software-Defined Networking
WSNs	Wireless Sensor Networks
SACFIR	SDN-Based Application-aware Centralized adaptive Flow Iterative Reconfiguring
SAMCFIR	SDN-Based Application-aware Main-value Centralized adaptive Flow Iterative Reconfiguring
MEMS	Micro Electro Mechanical System
FECHs	Forwarding Elements Cluster-Heads
non-FECHs	non-Forwarding Elements Cluster-Heads
SDCSN	Software Defined Clustered Sensor Networks
NMS	Network Management System
SOF	Sensor OpenFlow
IoT	Internet of Thing
SDWN	Software Defined Wireless Network
ATCEEC	Application-aware threshold-based Centralized Energy Efficient Clustering
MCEEC	Multi-hop Centralized Energy Efficient Clustering
SDN-IRWSNs	SDN-enabled Iteratively Reconfigurable WSNs
MCU	Micro Controller Unit
INP	Inter-Networking Processing
ASH	Average Surface Heights
LLDP	Link Layer Discovery Protocol
NTMP	Network Topology Management Phase
NFP	Network Forwarding Phase
NSP	Network Settling Phase
MCF	Minimum Cost Function

## References

[1] Heinzelman W., Chandrakasan A., Balakrishnan H. (2000). Energy-efficient communication protocol for wireless microsensor networks. Proceedings of the 33rd Annual Hawaii International Conference on System Sciences.

[2] Heinzelman W., Chandrakasan A., Balakrishnan H. (2002). An application-specific protocol architecture for wireless microsensor networks. IEEE Trans. Wirel. Commun..

[3] De Gante A., Aslan M., Matrawy A. (2014). Smart wireless sensor network management based on software-defined networking. Proceedings of the 2014 27th Biennial Symposium on Communications (QBSC).

[4] Garcia F.P., de Souza J.N., Andrade R.M. (2012). An energy-efficient passive monitoring system for wireless sensor networks. Proceedings of the Sustainable Internet and ICT for Sustainability (SustainIT).

[5] Galluccio L., Milardo S., Morabito G., Palazzo S. (2015). SDN-WISE: Design, prototyping and experimentation of a stateful SDN solution for WIreless SEnsor networks. Proceedings of the 2015 IEEE Conference on Computer Communications (INFOCOM).

[6] Luo T., Tan H.P., Quek T.Q. (2012). Sensor OpenFlow: Enabling software-defined wireless sensor networks. IEEE Commun. Lett..

[7] Keller L., Atsan E., Argyraki K., Fragouli C. (2013). SenseCode: Network coding for reliable sensor networks. ACM Trans. Sens. Netw..

[8] Gupta A., Vanbever L., Shahbaz M., Donovan S.P., Schlinker B., Feamster N., Rexford J., Shenker S., Clark R., Katz-Bassett E. (2015). SDX: A software defined internet exchange. ACM SIGCOMM Comput. Commun. Rev..

[9] Agarwal S., Kodialam M., Lakshman T. Traffic engineering in software defined networks. Proceedings of the 2013 IEEE INFOCOM.

[10] Yu H., Jia Z., Ju L., Liu C., Ding X. (2016). Energy Efficient Routing Algorithm Using Software Defining Network for WSNs via Unequal Clustering. Proceedings of the International Conference on Geo-Informatics in Resource Management and Sustainable Ecosystems.

[11] Kahjogh B.O., Bernstein G. (2017). Energy and latency optimization in software defined wireless networks. Proceedings of the 2017 Ninth International Conference on Ubiquitous and Future Networks (ICUFN).

[12] Bera S., Misra S., Roy S.K., Obaidat M.S. (2016). Soft-WSN: Software-Defined WSN Management System for IoT Applications. IEEE Syst. J..

[13] Li M., Zhao L., Liang H. (2017). An SMDP-based Prioritized Channel Allocation Scheme in Cognitive Enabled Vehicular Ad Hoc Networks. IEEE Trans. Veh. Technol..

[14] Liu Y., Dong M., Ota K., Liu A. (2016). ActiveTrust: Secure and trustable routing in wireless sensor networks. IEEE Trans. Inf. Forensics Secur..

[15] Luo S., Dong M., Ota K., Wu J., Li J. (2015). A security assessment mechanism for software-defined networking-based mobile networks. Sensors.

[16] Javaid N., Aslam M., Ahmad A., Khan Z.A., Alghamdi T.A. MCEEC: Multi-Hop Centralized Energy Efficient Clustering Routing Protocol for WSNs. Proceedings of the 2014 IEEE International Conference on Communications (ICC).

[17] Javaid N., Aslam M., Djouani K., Khan Z.A., Alghamdi T.A. ATCEEC: A new energy efficient routing protocol for Wireless Sensor Networks. Proceedings of the 2014 IEEE International Conference on Communications (ICC).

[18] Galluccio L., Milardo S., Morabito G., Palazzo S. Reprogramming Wireless Sensor Networks by using SDN-WISE: A hands-on demo. Proceedings of the 2015 IEEE Conference on Computer Communications Workshops (INFOCOM WKSHPS).

[19] Anadiotis A.C.G., Galluccio L., Milardo S., Morabito G., Palazzo S. Towards a software-defined Network Operating System for the IoT. Proceedings of the 2015 IEEE 2nd World Forum on Internet of Things (WF-IoT).

[20] Kobo H.I., Abu-Mahfouz A.M., Hancke G.P. (2017). A Survey on Software-Defined Wireless Sensor Networks: Challenges and Design Requirements. IEEE Access.

[21] Xiong X., Hou L., Zheng K., Xiang W., Hossain M.S., Rahman S.M.M. (2016). Smdp-based radio resource allocation scheme in software-defined internet of things networks. IEEE Sens. J..

[22] Ejaz W., Naeem M., Basharat M., Anpalagan A., Kandeepan S. (2016). Efficient wireless power transfer in software-defined wireless sensor networks. IEEE Sens. J..

[23] Anadiotis A.C.G., Morabito G., Palazzo S. (2016). An SDN-Assisted Framework for Optimal Deployment of MapReduce Functions in WSNs. IEEE Trans. Mob. Comput..

[24] O’Shea D., Cionca V., Pesch D. (2015). The Presidium of Wireless Sensor Networks—A Software Defined Wireless Sensor Network Architecture. Proceedings of the International Conference on Mobile Networks and Management.

[25] Abdolmaleki N., Ahmadi M., Malazi H.T., Milardo S. (2017). Fuzzy topology discovery protocol for SDN-based wireless sensor networks. Simul. Model. Pract. Theory.

[26] Wang H., Li Y., Jin D., Hui P., Wu J. (2016). Saving energy in partially deployed software defined networks. IEEE Trans. Comput..

[27] Olivier F., Carlos G., Florent N. (2015). SDN based architecture for clustered WSN. Proceedings of the 2015 9th International Conference on Innovative Mobile and Internet Services in Ubiquitous Computing (IMIS).

[28] Hu F., Hao Q., Bao K. (2014). A survey on software-defined network and openflow: From concept to implementation. IEEE Commun. Surv. Tutor..

[29] De Oliveira B.T., Gabriel L.B., Margi C.B. (2015). TinySDN: Enabling multiple controllers for software-defined wireless sensor networks. IEEE Lat. Am. Trans..

[30] Cao C., Luo L., Gao Y., Dong W., Chen C. (2016). TinySDM: Software defined measurement in wireless sensor networks. Proceedings of the 15th International Conference on Information Processing in Sensor Networks.

[31] Hong S., Kim D., Ha M., Bae S. (2010). SNAIL: An IP-based wireless sensor network approach to the internet of things. Wirel. Commun. IEEE.

[32] Costanzo S., Galluccio L., Morabito G., Palazzo S. Software Defined Wireless Networks: Unbridling SDNs. Proceedings of the European Workshop on Software Defined Networking.

[33] Gude N., Koponen T., Pettit J., Pfaff B., Casado M., McKeown N., Shenker S. (2008). NOX: Towards an operating system for networks. ACM SIGCOMM Comput. Commun. Rev..

[34] Pakzad F., Portmann M., Tan W.L., Indulska J. (2014). Efficient topology discovery in software defined networks. Proceedings of the 2014 8th International Conference on Signal Processing and Communication Systems (ICSPCS).

[35] Krishnan S., Yegin A., Montavont N., Njedjou E., Veerepalli S. (2007). Link-Layer Event Notifications for Detecting Network Attachments. https://tools.ietf.org/html/rfc4957.

